# Suppression of *Propionibacterium acnes* Infection and the Associated Inflammatory Response by the Antimicrobial Peptide P5 in Mice

**DOI:** 10.1371/journal.pone.0132619

**Published:** 2015-07-21

**Authors:** Sunhyo Ryu, Hyo Mi Han, Peter I. Song, Cheryl A. Armstrong, Yoonkyung Park

**Affiliations:** 1 Department of Biomedical Science, Chosun University, Gwangju, Korea; 2 Department of Dermatology, University of Colorado Denver Anschutz Medical Campus, Aurora, Colorado, United States of America; 3 Research Center for Proteineous Materials, Chosun University, Gwangju, Korea; San Gallicano Dermatologic Institute, ITALY

## Abstract

The cutaneous inflammation associated with acne vulgaris is caused by the anaerobic bacterium *Propionibacterium acnes* through activation of the innate immune system in the skin. Current standard treatments for acne have limitations that include adverse effects and poor efficacy in many patients, making development of a more effective therapy highly desirable. In the present study, we demonstrate the protective effects of a novel customized α-helical cationic peptide, P5, against *P*. *acnes*-induced inflammatory responses *in vitro* and *in vivo*. Application of P5 significantly reduced expression of two inflammatory cytokines IL-8 and TNF-α in *P*. *acnes*-treated primary human keratinocytes, where P5 appeared to act in part by binding to bacterial lipoteichoic acid, thereby suppressing TLR2-to-NF-κB signaling. In addition, in a mouse model of acne vulgaris, P5 exerted both anti-inflammatory and antimicrobial effects against *P*. *acnes*, but exerted no cytotoxic effects against skin cells. These results demonstrate that P5, and perhaps other cationic antimicrobial peptides, offer the unique ability to reduce numbers *P*. *acnes* cells in the skin and to inhibit the inflammation they trigger. This suggests these peptides could potentially be used to effectively treat acne without adversely affecting the skin.

## Introduction

Acne vulgaris is a multifactorial inflammatory disease, the symptoms of which include comedones, papules, pustules, nodules, cysts and pilosebaceous inflammation, often leading to significant scaring and disfigurement of the face and upper trunk [1]. *Propionibacterium acnes (P*. *acnes)* plays a key role in the pathogenesis of acne. This ubiquitous gram-positive bacterium is part of the normal skin microflora, but is present in abnormally high numbers within pilosebaceous follicles in patients with acne [2]. It is widely accepted that inflammatory acne reflects the immune response of the host to *P*. *acnes*, which can stimulate the production of proinflammatory cytokines and chemokines (e.g., IL-8 and TNF-α) by immune system cells, thereby triggering the granulomatous reactions of inflammatory skin disease [3]. Consistent with that idea, *P*. *acnes* releases chemoactive factors that attract neutrophils, monocytes and lymphocytes [4], and acts via the toll-like receptor (TLR)2-to-NF-κB signaling pathway to stimulate production of pro-inflammatory cytokines, chemokines and adhesion molecules [5, 6].

Reduction of the numbers of *P*. *acnes* in the skin by acne medications correlates with clinical improvement [2]. However, treatment of acne vulgaris with commonly used antibiotics, including oral tetracycline and topical erythromycin and clindamycin, has increased the resistance of *P*. *acnes* to antibiotics and increased the likelihood of therapeutic failure [7]. Benzoyl peroxide (BPO), a lipophilic non-antibiotic antibacterial agent, is also highly effective against *P*. *acnes*; however, it has a high minimal inhibitory concentration (MIC: 150 μg/ml) [8, 9]. Oral isotretinoin, a potent oral retinoid reserved for the treatment of severe acne, is effective, but its use is limited by its many severe side effects [10]. Recently, a 5% dapsone gel (a synthetic sulfone) was shown to have some efficacy in the treatment of acne vulgaris [11], but potential hematologic toxicity limits its clinical utility [12]. It is thus clear that development of a safe therapeutic reagent that has no side effects but strong antibacterial activity would highly desirable for the treatment of acne. One promising approach is the use of antimicrobial peptides (AMPs), which efficiently kill microbial pathogens and modulate host immune responses [13].

CA is a 37-amino acid cationic AMP isolated from the caterpillar of the cecropia moth (*Hyalaphora cecropia*) [14], while MA is a 23-amino acid cationic AMP from the skin of the African clawed frog (*Xenopus laevis*) [15]. Both CA and MA exert strong antibacterial effects with little or no cytotoxicity affecting mammalian cells [14, 15]. A primary target for these peptides is the lipid bilayer of the bacterial cytoplasmic membrane, to which they bind and induce membrane permeabilization [16]. We recently reported a synthetic CA-MA hybrid analogue, P5, which was designed to increase the net positive charge and hydrophobicity of the peptide [17, 18]. P5 showed greatly increased antibacterial activity against both Gram-positive and Gram-negative bacteria, acting via a membranolytic mechanism, but no hemolytic or cytotoxic activity in mammalian cells. Notably, P5 has stronger antibacterial and antifungal activities than CA-MA against a variety of aerobic microbes, but has little or no hemolytic or cytotoxic activity against normal eukaryotic cells [17–20]. Moreover, it has not yet been demonstrated whether P5 has the ability to effectively prevent or treat inflammatory skin diseases such as acne. In the present study, therefore, our aim was to assess the potential utility of P5 as a therapeutic agent for the treatment of inflammatory acne vulgaris based on its potent antibacterial effects and its ability to suppress development of the inflammatory responses triggered by *P*. *acnes*. To do that, we tested the effects of P5 on inflammatory cytokine production in living *P*. *acnes*-infected human keratinocytes (HKs) *in vitro*, and investigated the molecular mechanism of the anti-inflammatory effects of P5 in a mouse model of *P*. *acnes*-induced inflammatory acne.

## Materials and Methods

### Ethics Statement

This study was carried out in strict accordance with the recommendations in the Guide for the Care and Use of Laboratory Animals of the National Institutes of Health. The protocol was approved by the Committee on the Ethics of Animal Experiments of the University of Arkansas for Medical Sciences (Permit Number: A3063-01). All surgery was performed under sodium pentobarbital anesthesia, and all efforts were made to minimize suffering.

### Experimental animal protocol

We utilized female wild type Institute of Cancer Research (ICR) mice (6–8 weeks-old; Harlan, Indianapolis, IN) in this study as a means of assessing the ability of the newly designed synthetic antimicrobial peptide P5 to minimize a *Propionibacterium acnes* (*P*. *acnes*) infection, and modulate the innate inflammatory response of the host as described in detail previously [21]. Birefly, P5 (1.6 μM, 20 μl), or PBS (20 μl) was injected intradermally into the right ears of ICR mice 24 hours after *P*. *acnes* (1 x 10^8^ CFU per 20 μl in PBS) inoculation at the same site. *P*. *acnes* was quantified by plating serial dilutions of the homogenate on agar plates and incubating under anaerobic conditions for 48 hours. Ear thickness was measured using a micro caliper (Mitutoyo 547-400S; MSI Viking Gage, Charleston, SC) prior to injection and at 24, 48, 72 and 96 hours after injection. For all *in vivo* experiments, n = 10 animals/treatment group/time point were used unless otherwise specified. Experimental comparisons were made by a repeated measures ANOVA model using SAS Proc mixed software (SAS Institute, Inc., Cary, NC).

### Preparation of synthetic peptides

Synthetic AMPs CA-MA (KWKLFKKIGIGKFLHSAKKF-NH2), P5 (KWKKLLKKPLLKKLLKKL-NH2), and P4 (KWKKKKKKPKFL-NH2) were synthesized as described previously [17]. The stock solution was made at a concentration of 1 mM in sterilized distilled water and filtered through a 0.22-μm pore filter. The filtered solution was stored at -20°C until use.

### Bacterial culture

*P*. *acnes* (ATCC 11828 and ATCC 6919)(American Type Culture Collection, Manassas, VA) was cultured in Reinforced Clostridial Medium (BD: Franklin Lakes, NJ) under anaerobic conditions using Gas-Pak at 37°C as described in detail previously [21]. *Staphylococcus epidermidis (S*. *epidermidis)* (ATCC 12228) was cultured in Nutrient broth (BD) under aerobic conditions at 37°C. For stimulation of normal human keratinocytes (HKs), bacterial cells were harvested by centrifugation at 3,000 x *g* for 10 min at 4°C. The bacteria were then washed three times with PBS and resuspended in starvation medium lacking hydrocortisone and bovine pituitary extract (BPE) or in PBS at 1x10^8^ colony-forming units (CFU)/ml.

### *In vitro* antibacterial assays

Minimum bactericidal concentrations (MBCs) of the peptides against anaerobic and aerobic microorganisms were determined in duplicate in two independent experiments using microdilution methods with 96-well microtitre cell culture plates. The peptides were filtered through 0.22 μm filters and diluted stepwise in appropriate broth media to concentrations ranging from 100 μM to 0.39 μM. The 2-fold serially diluted solutions of each peptide (100 μl) were mixed with 100 μl of bacterial suspension to a density of 2x10^6^ CFU/ml. The plates were then incubated for 16 h at 37°C under anaerobic or aerobic conditions. After incubation, the reaction mixtures were diluted and plated onto appropriate agar plates for counting the CFUs. The MBC was defined as the lowest peptide concentration that resulted in no visible microorganism growth on the agar plate.

### Keratinocyte culture

Human epidermal keratinocytes (HKs) from foreskin were purchased from PromoCell (Heidelberg, Germany) and cultured in keratinocyte growth medium (KGM; Clonetics, San Diego, CA) as described in detail previously [21]. Briefly, HK cells were cultured in KGM at 37°C in a humidified atmosphere containing 5% CO_2_. Cell culture media was changed every 2 to 3 days and cells were harvested after passage 3 to 4. HK cells were seeded at a concentration of 10^4^ to 10^5^ cells per well into 6/12/96 well culture plates and chamber slides. The cells were allowed to grow up to about 70% confluence. In the required experimental conditions, HK cells were cultured in KGM starving media, which is free of supplement and growth factors (BPE, hEGF, insulin, hydrocortisone, epinephrine, transferrin and gentamicin/amphotericin-B).

### Scanning electron microscopy (SEM)

SEM was performed as described previously [18]. All samples were visualized using a field emission-scanning electron microscope (FE-SEM, JSM-7100F, Jeol, Japan) at 20,000x magnification (15.0 kV).

### Treatment of *P*. *acnes* and/or peptides in HKs

After cultured HKs were pre-infected with *P*. *acnes* (1x 10^8^ CFU/ml) for 24 hours, a synthetic peptide (CA-MA, P5 or P4) was added to each well to a concentration of 0.8 μM, 1.6 μM or 3.2 μM. Then, the cells were incubated for various periods as indicated in the results. Cultured HK cells in starvation medium only served as a negative control. The cells were then harvested for RNA extraction. The supernatants collected by centrifugation at 14,000 x *g* for 10 min at 4°C were aliquoted and stored at -70°C until used in IL-8 and TNF-α assay.

### Quantitative RT-PCR

Target gene mRNA expression was analyzed by real-time RT-PCR as described in the manufacturer’s protocol (ABI 7500 real-time PCR system using SYBR Green master mix; Applied Biosystems, Foster City, CA) as described in detail previously [21]. The primer sequences were as follows: for IL-8, 5'-GCAGTTTTGCCAAGGAGTGCT-3' (sense) and 5'-TTTCTGTGTTGGCGCAGTGTG-3' (antisense); for TNF-α, 5’-ATAGCTCCCAGAAAAGCAAGC-3’ (sense) and 5’-CACCCCGAAGTTCAGTAGACA-3’ (antisense); for TLR2, 5’-TGTCTTGTGACCGCAATGGT-3’ (sense) and 5’-GTTGGACAGGTCAAGGCTTT-3’ (antisense); and for 18S rRNA, 5'-CGGCTACATCCAAGGAA-3' (sense) and 5'-GCTGGAATTACCGCGGCT-3' (antisense). Quantification of target gene expression was normalized using an internal control gene, 18S rRNA [22]. All the experiments were performed in triplicate.

### Enzyme-Linked Immunosorbent Assay (ELISA)

The IL-8 and TNF-α levels in collected culture supernatants were determined using human TNF-α and IL-8 immunoassay kits (R&D Systems, Minneapolis, MN) according to the manufacturer’s instructions. The optical density of the wells was measured using an ELISA reader set to 450 nm with a wavelength correction set to 540 nm. All the experiments were performed in triplicate.

### Immunofluorescent staining for NF-κB nuclear translocation and TLR2 cellular localization

Immunofluorescence analysis of NF-κB and TLR2 localization was performed as previously described [23]. Briefly, HKs were treated with *P*. *acnes* (1 x 10^8^ CFU/ml) for 30 min for NF-κB or 24 h for TLR2 in the presence or absence of 1.6 μM P5 or P4. The cells were then first incubated with rabbit polyclonal anti-human NF-κB p65 antibody (Rel A) or rabbit anti-human TLR2 antibody (Rockland, Gilbertsville, PA), diluted 1:3000 in blocking buffer (ImmPRESS kit; Vector Laboratories, Burlingame, CA). They were then incubated for 1 h with FITC-conjugated affinity-purified goat anti-rabbit IgG (1:300 dilution; H+L; Jackson ImmunoResearch Laboratories, Inc., West Grove, GA) at room temperature in the dark. Finally, the cells were visualized under a microscope (Olympus EX51; Center Valley, PA), and images were acquired using a QICAM fast 1394 camera (Westmont, IL).

### MTT assay

A standard colorimetric assay for assessing cell viability based on the activity of MTT (yellow tetrazolium salt: 3-(4,5-dimethuylthiazol- 2-yl)-2,5-diphenyltetrazolium bromide) reducing enzymes was performed according to the manufacturer’s instructions (Molecular Probes, Inc., Eugene, OR) using HK cells (5 x 10^3^ per 200 μl culture media) in the presence and absence of P5 or P4 at concentrations ranging from 1.6 to 6.4 μM. Data are presented as the percentage of viable HK cells compared to the percentage of viable cells after treatment with 2% Triton X-100, which served as the positive control for cell cytotoxicity.

### Circular dichroism (CD) analysis of P5 binding to lipoteichoic acid (LTA)

CD spectra were recorded at 25°C on a Jasco 810 spectropolarimeter (Jasco, Tokyo, Japan) equipped with a temperature control unit using a quartz cell with a 0.1-cm path length. The CD spectra for 50 μM peptide dissolved in PBS (pH 7.2) were scanned in the presence or absence of 0.1% LTA dissolved in PBS. At least four scans of the 250–190 nm wavelength range were conducted, after which the average blank spectra were subtracted from the average of the sample spectra.

### Analysis of intracellular Ca^2+^ mobilization in keratinocytes

Mobilization of intracellular Ca^2+^ was evaluated to assess the functional response of HKs to *P*. *acnes*-infection in the presence and absence of synthetic peptides as described in detail previously [21]. Briefly, cultured HKs on cover slips were incubated for 45 min at 37°C in PBS containing 2 μM fura-2/AM (Invitrogen, Carlsbad, CA), the membrane permeant form of the fluorescent Ca^2+^ probe fura-2. After three washes, the cells were pretreated with or without 1.6 μM P5 or P4, after which *P*. *acnes* was added during the active monitoring of intracellular Ca^2+^ mobilization. Fluorescence was measured using an InCyt Basic IM Fluorescence Imaging System (Intracellular Imaging Inc., Cincinnati, OH) according to the manufacturer’s instructions. The intracellular free Ca^2+^ concentration was determined by measuring the ratio of the 510 nm emissions elicited by excitation at 340 and 380 nm.

### Determination of *P*. *acnes* abundance and *P*. *acnes*-induced inflammation *in vivo*

P5 (1.6 μM, 20 μl) or PBS (20 μl) was intradermally injected into the right ears of ICR mice (Harlan, Indianapolis, IN) 24 h after *P*. *acnes* (1 x 10^8^ CFU per 20 μl in PBS) inoculation at the same site. Left ears of the same mice were injected with 20 μl of PBS. In negative control ICR mice, the right ears remained untreated, while the left ears received intradermal injections of PBS. Ten mg of tissue from 8 mm punch biopsies taken from the ears 24 hours after peptide injection were homogenized in 250 μl of sterile PBS using a tissue grinder. *P*. *acnes* was quantified by plating serial dilutions of the homogenate on agar plates and incubating them for 48 h under anaerobic conditions. Ear thickness was measured using a micro caliper (Mitutoyo 547-400S; MSI Viking Gage, Charleston, SC) prior to peptide injection and at 24, 48 and 72 hours after injection.

### Statistical analysis

Results are expressed as means ± SD. ANOVA with probabilities was performed for both overall significance and pairwise comparison. *P*<0.05 was considered to be statistically significant.

## Results

### Antibacterial effects of CA-MA, P5 and BPO against skin bacteria

We compared the antibacterial activities of CA-MA, its newly designed analogue P5, and BPO against the skin bacteria *P*. *acnes ATCC* 11828, *P*. *acnes ATCC* 6919 and *S*. *epidermidis* ATCC 12228 by determining the MBC of each strain using the microdilution method. The three strains were cultured in the presence of various concentrations each agent for 48, 72 and 24 hours, respectively. Bacterial growth was then evaluated by measuring the absorbance at 600 nm, after which the bacteria were diluted and spotted on agar plates to count the CFUs (Table 1). Each synthetic peptide, CA-MA (parental peptide), P5 and a negative control peptide (P4), was tested at concentrations ranging from 0.1 to 12.8 μM using twofold serial dilutions. BPO, which is a traditional compound used to treat mild to moderate acne, was tested at concentrations ranging from 7.8 to 250 μM, with 5% (vv-1) DMSO serving as the control. The MBC values of P5 and CA-MA were 0.2 and 0.4 μM, respectively (Table 1). The MBC for P5 was 300 times lower than that for BPO (62.5 μM) and 64 times lower than for P4 (>12.8 μM), its negative control peptide. The potent antibacterial activity of P5 against anaerobic *P*. *acnes* is similar to that of clindamycin (MBC <0.2 μM), a common topical treatment for acne vulgaris. These results demonstrate that increasing the positive charge and the hydrophobicity of the newly designed AMP P5 led to a distinct increase in its bactericidal activity.

**Table 1:** MBCs of Synthetic AMPs and Conventional Agents against Acne-Inducing *Propionibacterium acnes*.

Antimicrobial peptides (AMPs)	MBC (μM) of the following bacteria		
	*P*. *acnes (ATCC11828)*	*P*. *acnes (ATCC6919)*	*P*. *acnes (ATCC12228)*
**CA-MA**	0.4	0.4	0.4
**P5**	0.2	0.2	0.2
**P4**	>12.8	>12.8	>12.8
**Clindamycin**	<0.2	<0.2	<0.2
**BPO (benzoyl peroxide)**	62.5	>62.5	>62.5

MBC (minimal bactericidal concentration) was defined as the lowest peptide concentration (μM) that prevented 100% of bacterial growth on agar plates.

### Cytotoxicity of P5 against HKs

To determine the cytotoxic effects of P5 on HKs, we used MTT assays to measure HK viability in serum-free medium with or without P5 for 24 h at 37°C. HK viability was determined after 24 h of treatment at concentrations ranging from 0.5 to 5 μM. We found that HKs were 100% viable after 24 h of treatment with P5 or its control, P4 (S1 Table). These results demonstrate that P5 has no significant cytotoxic effect on HKs, even at concentrations several times higher than necessary for antibacterial activity against *P*. *acnes*.

### P5 induces morphological disruption of *P*. *acnes* that suggests a bactericidal effect

**Fig 1:**
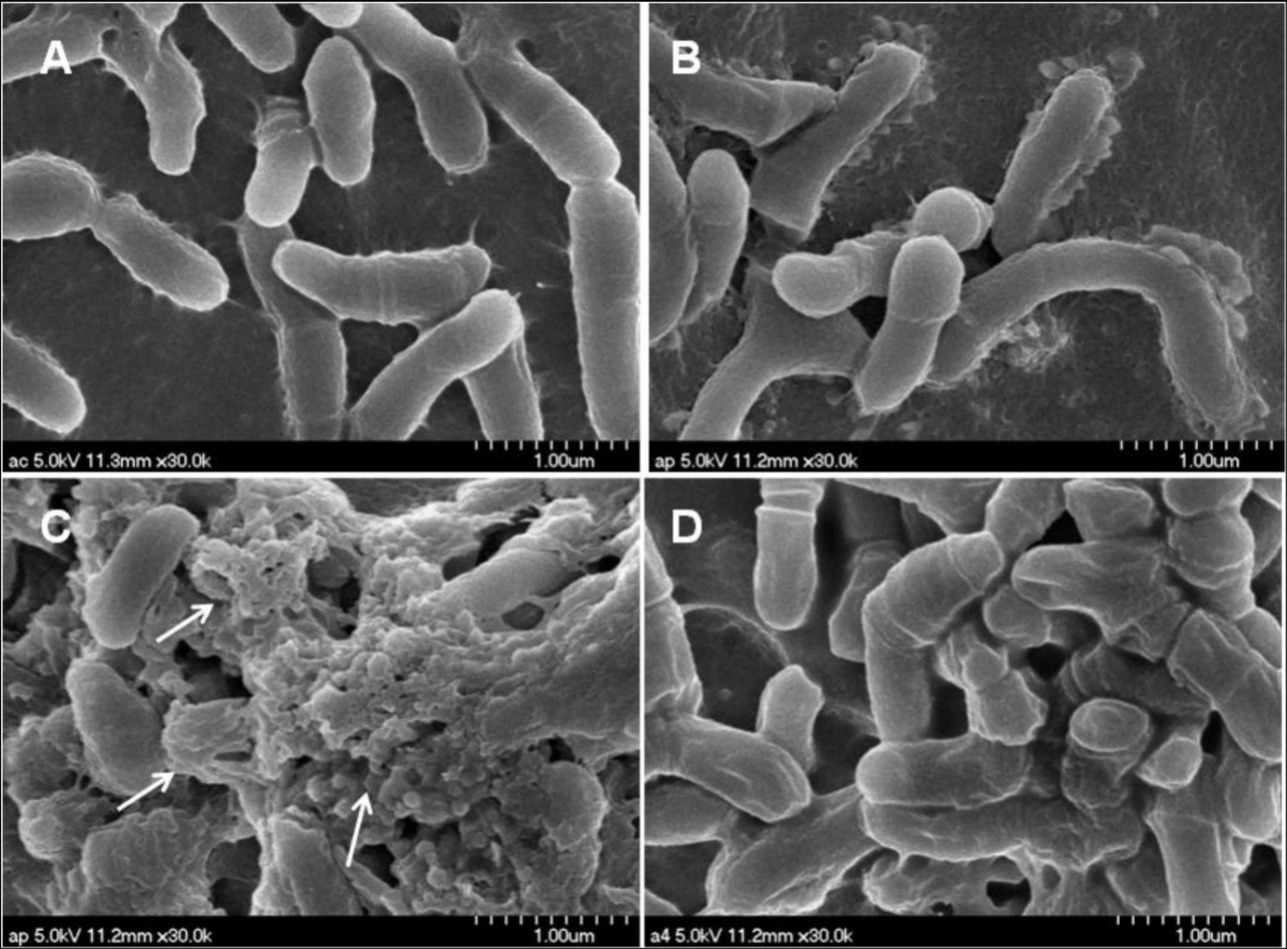
Morphological perturbations and blebs induced in *P*. *acnes* by P5. Shown are *P*. *acnes* cells after incubation for 20 min in the absence (A) and presence of CA-MA (B), P5 (C) and P4 (D) at a concentration of 1/2 MBC. Arrows point to morphological perturbations and blebs, which were clearly visible following treatment with P5.

Cationic AMPs kill bacteria primarily through membrane permeabilization and subsequent structural disruption. Using SEM, we were able to directly observe cell morphology and integrity after peptide treatment. We observed that P5 induced morphological perturbations and blebs in the *P*. *acnes* cell wall at a concentration of 0.1 μM (Fig 1C), whereas CA-MA (Fig 1B) and P4 (Fig 1D) induced little morphologic changes to the cell surface at the same concentration. These results indicate that the antimicrobial effect of P5 against *P*. *acnes* is consistent with its cationic nature with its bactericidal or bacteriostatic activity.

### P5 suppresses *P*. *acnes*-induced expression of pro-inflammatory cytokines in HKs

**Fig 2:**
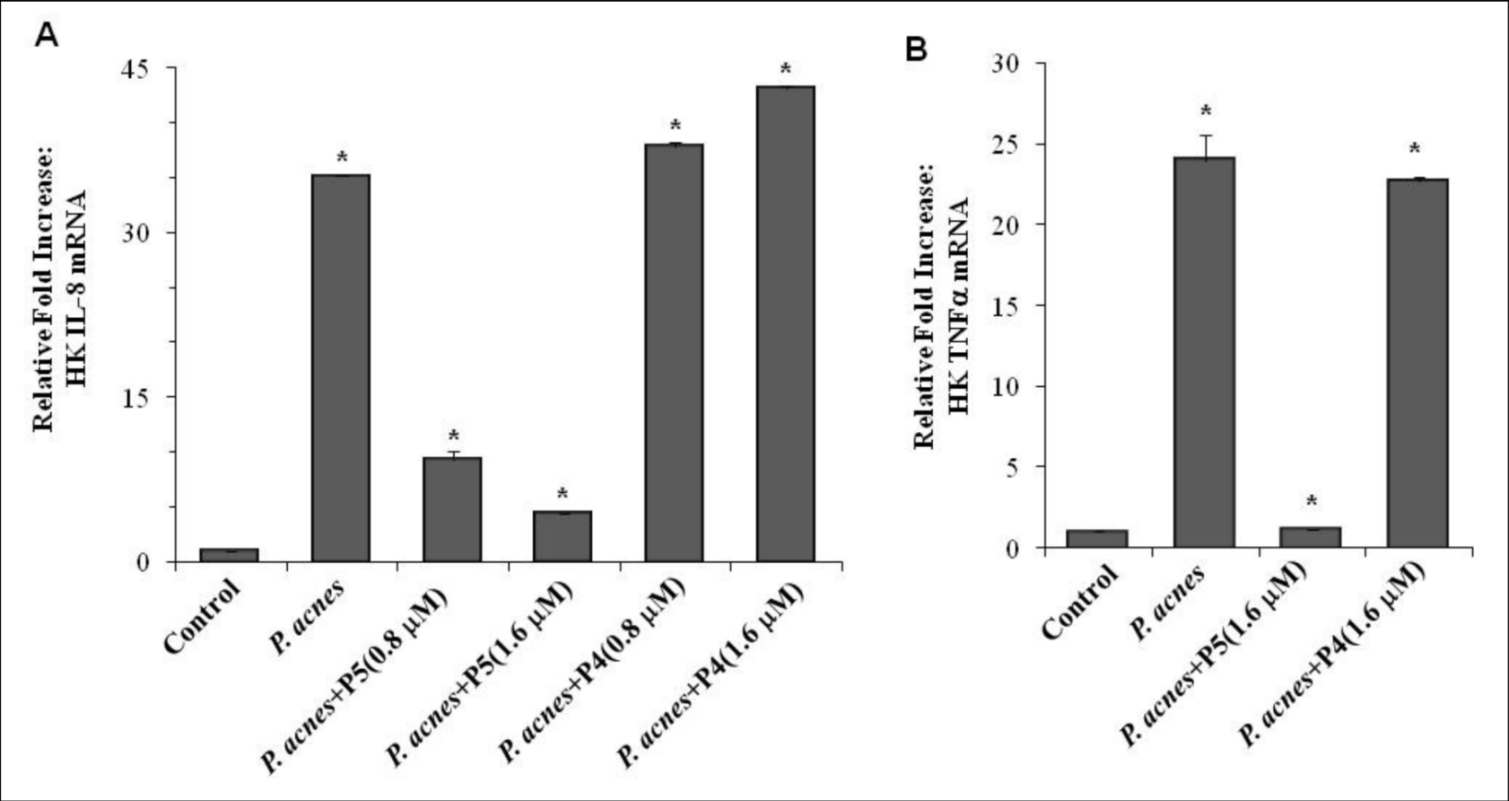
P5 inhibits expression of IL-8 and TNF-α mRNA induced by *P*. *acnes* infection in HKs. Total RNA was collected from *P*. *acnes*-infected HKs with and without P5 treatment. Expression of IL-8 (A) and TNF-α (B) mRNA was measured using quantitative RT-PCR with human IL-8- and TNF-α-specific primers. The relative level of each mRNA was normalized to the expression of 18S rRNA. All data were compared to the untreated control values. The data shown are representative of triplicate experiments. All values are expressed as the mean ± SD. *p<0.001.

**Fig 3:**
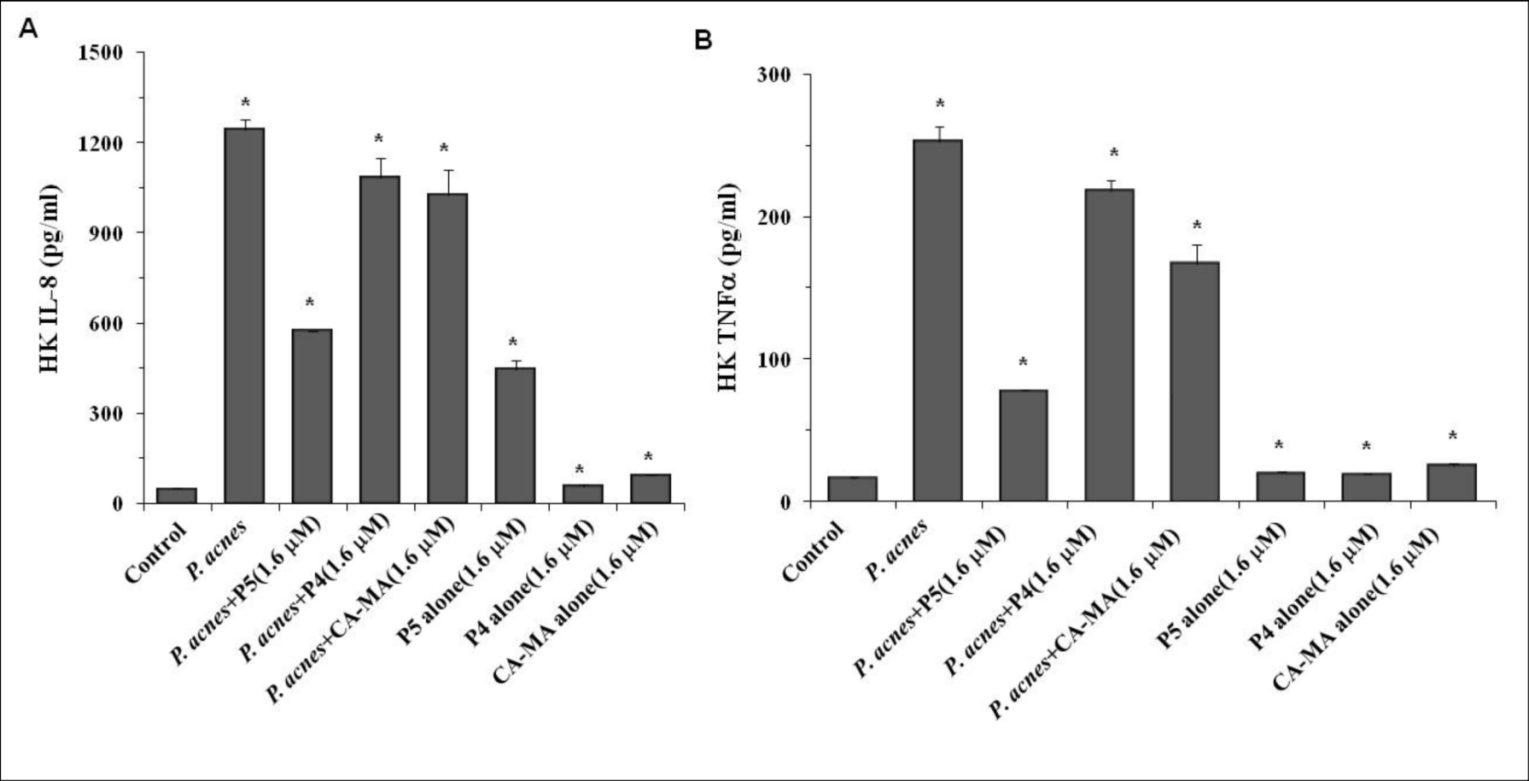
*P*. *acnes*-induced secretion of IL-8 and TNF-α from HKs was inhibited by P5 treatment. IL-8 (A) and TNF-α (B) were measured in culture supernatants after incubating *P*. *acnes*-infected HK cells for 24 h in the presence or absence of 1.6 μM P5, P4 or CA-MA. The data shown are representative of triplicate experiments. All values are expressed as mean ± SD. *p< 0.001.

We next examined the effect of P5 on innate inflammatory responses to *P*. *acnes* infection *in vitro*. Because IL-8 and TNF-α are inflammatory mediators likely to participate in the response to cutaneous infections, we used real-time RT-PCR (Fig 2) and ELISA (Fig 3) to measure the effects of P5 (0.8 or 1.6 μM) on the expression of IL-8 and TNF-α 24 h after pre-infection (1x10^8^ CFU/ml) of HKs with *P*. *acnes*. We found that relative levels of both IL-8 and TNF-α mRNA (Fig 2A and 2B, respectively) and protein (Fig 3A and 3B respectively) were significantly upregulated after *P*. *acnes* infection, and those responses were significantly inhibited by P5. Parental CA-MA had much less ability to inhibit the increase in IL-8 and TNF-α expression induced by *P*. *acnes* than the more hydrophobic P5 analogue, while the negative control peptide P4 had no effect on *P*. *acnes*-induced IL-8 and TNF-α expression in HKs. By contrast, these AMPs had no effect on basal IL-8 or TNF-α expression when uninfected HKs were treated (Fig 3A and 3B). These results indicate that P5 specifically inhibits the expression of inflammatory cytokines induced by *P*. *acnes* infection in HKs.

### P5 significantly inhibits *P*. *acnes*-induced TLR2 expression in HK cells

**Fig 4:**
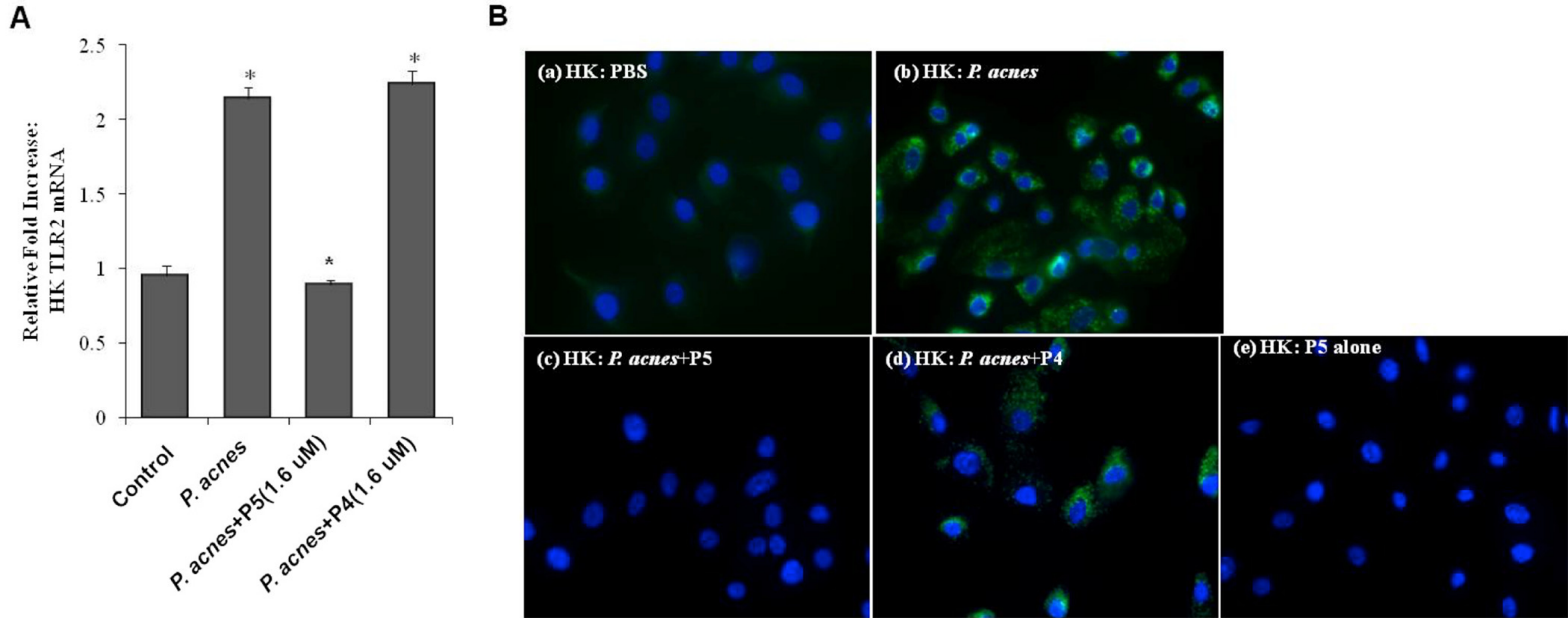
P5 inhibits expression of TLR2 induced by *P*. *acnes* in HKs. (A) Expression of TLR2 mRNA was measured using real-time RT-PCR and normalized to the expression of 18S rRNA. All values are expressed as the mean ± SD. *P<0.001. (B) Immunofluorescent localization of TLR2 within HKs. *P*. *acnes*-infected HKs were incubated for 24 h in the presence or absence of 1.6 μM P5 or P4, after which the distribution of TLR2 was determined by immunofluorescent labeling. FITC-labeled TLR2 is shown in green, while the Hoechst-stained nuclei are blue: (a) treated with PBS; (b) treated with *P*. *acnes*; (c) treated with *P*. *acnes* plus P5; (d) treated with *P*. *acnes* plus P4; (e) treated with P5 alone.

It is known that *P*. *acnes* contributes to inflammation in acne through activation of the toll-like receptors (TLRs), especially TLR2 [19]. Therefore, to further explore the effect of P5 on innate inflammatory responses, we used real-time RT-PCR to assess expression of the pattern recognition receptor TLR2 induced in HKs in response to *P*. *acnes* (1x10^8^ CFU/ml) infection and then tested the effect of 1.6 μM P5. Expression of TLR2 mRNA was increased about 2-fold in HKs 24 h after *P*. *acnes* inoculation, and this overexpression was significantly (*P*<0.001) down-regulated by treatment with P5, but not P4 (Fig 4A). P5 also inhibited *P*. *acnes*-induced expression of TLR2 protein in HKs measured 24 h after treatment (Fig 4Bc), as compared to the negative control P4 (Fig 4Bd). Finally, treatment with P5 alone (Fig 4Be) had no effect on basal TLR2 expression in HKs. These results demonstrate that P5 specifically inhibits TLR2 expression related to the innate immune response to *P*. *acnes*-infection in HK cells.

### P5 blocks NF-κB nuclear translocation induced by *P*. *acnes* infection in HK cells

**Fig 5:**
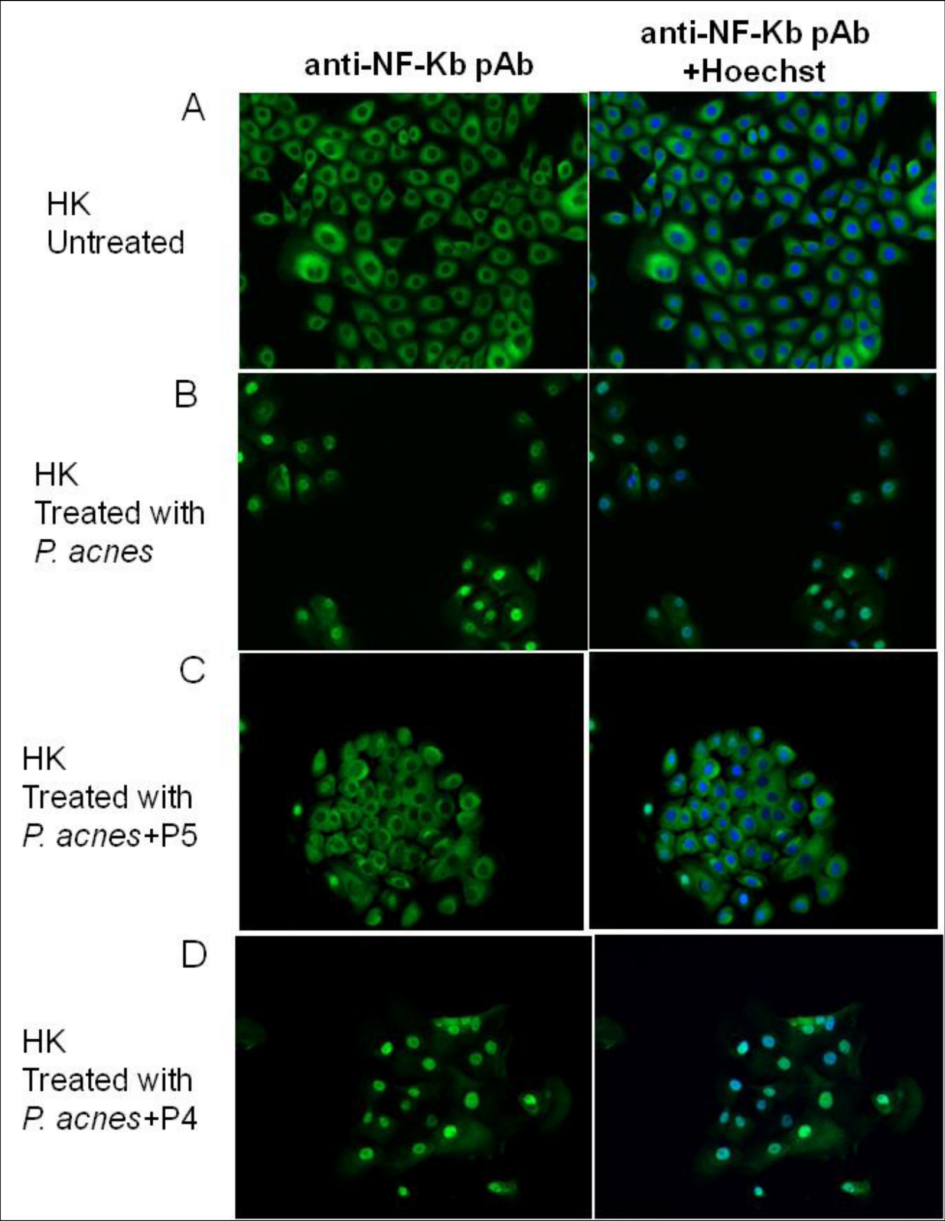
P5 inhibits NF-κB nuclear translocation in *P*. *acnes*-infected HK cells. The intracellular distribution of NF-κB was determined by immunofluorescent labeling of NF-κB p65 (green), while nuclei were Hoechst stained (blue): (A) untreated HK cells; (B) HK cells treated with *P*. *acnes*; (C) HK cells treated with *P*. *acnes* plus P5; HK cells treated with *P*. *acnes* plus P4 (D).

NF-κB is a key transcriptional regulator of multiple genes, including IL-8 and TNF-α. TLR2 signaling leads to activation of the NF-κB pathway, which in turn stimulates release of pro-inflammatory cytokines such as IL-8 and TNF-α. To test whether P5 affects TLR signaling to NF-κB, we used immunofluorescent staining to assess the intracellular distribution of NF-κB p65. In untreated HKs, NF-κB staining was observed primarily in the cytoplasm (Fig 5A), however, nuclear translocation of NF-κB was rapidly induced by *P*. *acnes* infection (Fig 5B). Co-incubation of HKs with *P*. *acnes* and P5 effectively blocked *P*. *acnes*-induced NF-κB nuclear translocation (Fig 5C). By contrast, the negative control peptide P4 had no effect on *P*. *acnes*-induced NF-κB nuclear translocation (Fig 5D).

### Binding of CA-MA and P5 to LTA

The ability of AMPs to bind LTA likely plays a significant role in their ability to neutralize LTA shed from bacteria and thus prevents inflammatory responses. We therefore analyzed the peptides’ CD spectra to ask whether any of the peptides studied here could bind to purified LTA *in vitro*. When the peptides were studied at 50 μM in a 0.1% LTA suspension, P5 adopted a more folded conformation than CA-MA (S1 Fig.), which is indicative of its binding to LTA. This suggests that P5 blocks the induction of inflammatory cytokines in HK cells by binding LTA.

### P5 inhibits *P*. *acnes*-induced intracellular Ca^2+^ fluctuation in HKs

**Fig 6:**
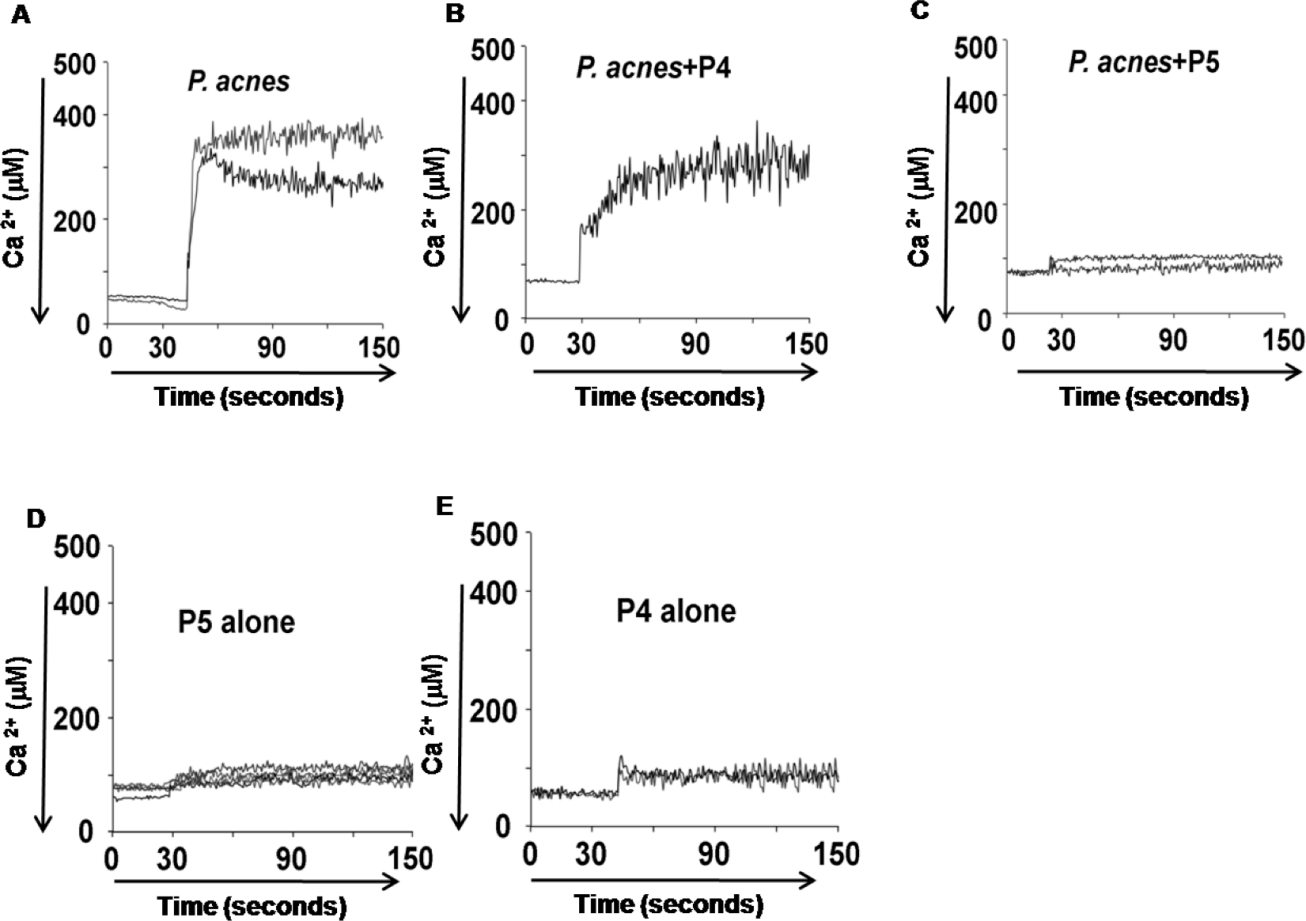
P5 inhibits *P*. *acnes*-induced intracellular Ca^2+^ mobilization in HK cells. Fura-2-loaded HKs on glass coverslips were treated with *P*. *acnes* (1 x 10^8^ CFU/150 μl) in the presence or absence of 0.8 μM P5 or P4. Uninfected HKs treated with P5 and P4 served as negative controls. The intracellular free Ca^2+^ concentration was determined by measuring the ratio of fura-2 fluorescence at 510 nm elicited by excitation at 340 and 380 nm. The peaks in this figure represent the simultaneous intracellular Ca^2+^ responses of different cells to *P*. *acnes* or the indicated AMP.

Ca^2+^ signaling appears to play a role at several steps during bacterial infection and in the control of gene expression, especially the expression and secretion of pro-inflammatory mediators induced by bacterial pathogens [19]. We therefore examined the effect of P5 on the intracellular Ca^2+^ signaling induced in HKs by *P*. *acnes* infection. As shown in Fig 6, *P*. *acnes* (1x10^8^ CFU/150 μl) infection led to a rapid intracellular Ca^2+^ response that was significantly abrogated by addition of P5 (0.8 μM) 1 h before *P*. *acnes* infection (Fig 6A and 6C). By contrast, when HKs were preincubated with P4, the negative control peptide, no inhibition of intracellular Ca^2+^ fluctuation was observed (Fig 6B). Stimulation with P5 or P4 alone had no effect on basal Ca^2+^ signaling in HKs (Fig 6D and 6E). These results indicate that P5 efficiently blocks the rapid increase in intracellular free Ca^2+^ induced in HKs by *P*. *acnes* infection.

### Inhibitory effects of P5 on *P*. *acnes* growth and induction of inflammatory responses *in vivo*

**Fig 7:**
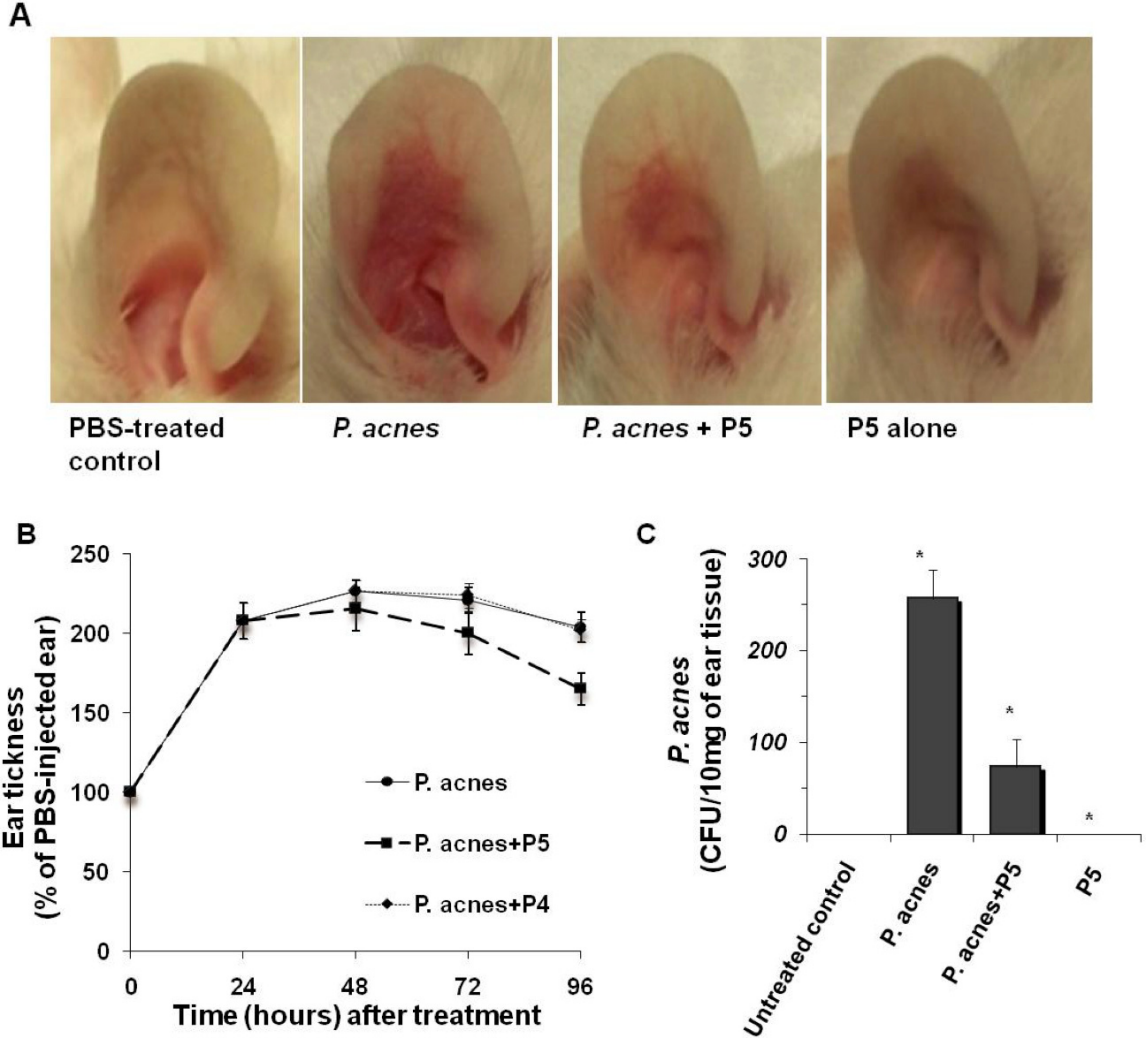
Effects of intradermal injection of P5 on *P*. *acnes* cell growth and *P*. *acnes*-induced inflammation in ICR mice. (A) Inflammation-associated erythema was visualized 24 h after injection of live *P*. *acnes* (1x10^8^ CFU/20 μl in PBS), *P*. *acnes* plus P5 (1.6 μM) or P5 alone into the ears of ICR mice. (B) Percent differences (right *vs*. left (control) ear) in ear edema compared among treatment groups every 24 h for 96 h. (C) Total number of *P*. *acnes* (CFUs) recovered from the ears of mice in the indicated treatment groups. All values represent mean ± SD of three individual experiments (*P<0.001). Untreated, uninfected ears served as a negative control.

To induce an *in vivo* model of *P*. *acnes* inflammation, we injected 10^8^ CFU of live *P*. *acnes* intradermally into the ears of ICR mice. After 24 h, the animals exhibited edema and severe cutaneous erythema, typical symptoms of ear inflammation, not seen in the negative control ears injected with PBS (Fig 7A). In the absence of *P*. *acnes* infection, untreated ears, P5-injected ears and P4-injected ears all served as negative controls. P5 alone had no effect on ear inflammation, as compared to untreated ears. Following inoculation of *P*. *acnes*, intradermal injection of P5 effectively reduced the erythema otherwise seen in the infected ears (Fig 7A). The reduction in inflammation after treatment was quantified by measuring ear thicknesses using a microcaliper. *P*. *acnes* inoculation led ears to swell to 2 times their original width within 24 h (Fig 7B). No swelling was observed in negative control ears injected only with PBS, and ear swelling was significantly reduced when mice were administered P5 (Fig 7B). Furthermore, P5 significantly reduced the number of *P*. *acnes* CFUs retrieved from the ear tissue (Fig 7C). Thus intradermal P5 injection exerts antibacterial effects against *P*. *acnes in vivo* and suppresses the inflammation otherwise seen in response to *P*. *acnes* infection.

## Discussion

The pathogenesis of acne vulgaris involves follicular hyperkeratinization, sebum production, the presence and activity of *P*. *acnes*, and inflammation [4, 24]. Although *P*. *acnes* is part of the normal human cutaneous flora, it is thought that colonization and proliferation by this organism plays a major role in the development of inflammatory acne lesions. The bacterial colonization is preceded by hyperproliferation of keratinocytes and increased sebum secretion in a hair follicle [4, 25]. Increased numbers of *P*. *acnes* are commonly found within the follicles of acne patients, and reductions in those counts correlates with clinical improvement [26]. Accordingly, antimicrobial agents and antibiotics (e.g., clindamycin, erythromycin and BPO) have been the mainstay of acne therapies targeting *P*. *acnes* colonies. However, use of topical and systemic antibiotics is complicated by the development of resistant strains of *P*. *acnes* [27].

In human cells, the plasma membrane is mainly composed of uncharged or zwitterionic lipids, whereas bacterial membranes are composed of various anionic components, including peptidoglycan, the phospholipids phosphatidylglycerol and cardiolipin, lipid A and teichoic acids, which give them a net negative charge [28]. With its net positive charge, P5 exhibits strong, selective affinity for the anionic surfaces of bacteria. Cationic AMPs, like P5, show broad-spectrum antimicrobial activity, kill target cells rapidly, and are active against antibiotic resistant and clinically relevant pathogens, including *P*. *acnes* [3, 29, 30]. It is generally accepted that the mechanism of action of cationic AMPs involves their direct interaction with the negatively charged bacterial membranes, which causes an increase in membrane permeability that rapidly leads to cell death [16]. Moreover, recent studies have shown that AMPs also exhibit direct anti-inflammatory activity [31] and inhibit the inflammation triggered by bacteria [32]. Indeed, several of these cationic peptides have shown potential for use in the treatment of acne vulgaris [33], which has both bacterial and inflammatory components.

In the present study, we show for the first time that P5 more strongly inhibits the growth of skin bacteria, including anaerobic *P*. *acnes* (ATCC 11828 and ATCC 6919) and aerobic *S*. *epidermidis* (ATCC 12228), than does BPO, an oxidizing agent frequently used to treat acne, or the CA-MA parent peptide (Table 1). Our findings further indicate that the positive charge and hydrophobicity of P5 are important determinants of its bactericidal activity. The susceptibility of *P*. *acnes* (ATCC 11828 and ATCC 6919) to synthetic cationic peptides suggests a potential for their use in developing topical treatments for acne vulgaris. Importantly in that regard, when P5 was incubated for 24 h with normal HKs, it showed no cytotoxicity at concentrations exerting antimicrobial activity (S1 Table). Taken together, these findings suggest that P5 specifically disrupts the bacterial cell surface without affecting mammalian cell membranes.

It is well established that *P*. *acnes* plays a pivotal role in the development of inflammatory skin diseases [2]. Several studies have shown that infection with *P*. *acnes* involves an interaction with TLR2 and TLR4 on keratinocytes [34]. Activation of these pattern recognition receptors induces release of inflammatory cytokines and chemokines, including TNF-α and IL-8 [3], which mediate inflammatory responses in both keratinocytes and monocytes [35]. Both TNF-α and IL-8 reportedly exacerbate skin inflammation in mice [36], and both lipopolysaccharide (LPS) and *P*. *acnes* directly stimulate the production of TNF-α and IL-8 via TLR expression [37]. Recent studies have shown that cationic peptides can inhibit the inflammation induced by bacteria, perhaps by altering cytokine production [30–32]. For example, several synthetic cationic AMPs have been shown to block LPS- and LTA-stimulated production of pro-inflammatory cytokines by macrophages [29]. In addition, Nagaoka *et al*, demonstrated that CAP11, an 11-kDa cationic AMP isolated from guinea pig neutrophils, inhibits the production of IL-8 and TNF-α in LPS-treated human peripheral blood mononuclear cells [38], while Mclnturff *et al*. reported that granulysin and synthetic granulysin-derived peptides downregulate the production of inflammatory cytokines and chemokines, including IL-8, in *P*. *acnes*-stimulated monocytes [39]. Our results show that P5 effectively inhibits the mRNA expression and protein secretion of both IL-8 and TNF-α in *P*. *acnes*-treated primary HKs, even though the cells were infected with *P*. *acnes* for 24 h prior to P5 treatment (Figs 2 and 3). These results demonstrate that the anti-inflammatory activity of P5 may be attributable to its ability to inhibit host-cell secretion of proinflammatory cytokines such as TNF-α and IL-8.

**Fig 8:**
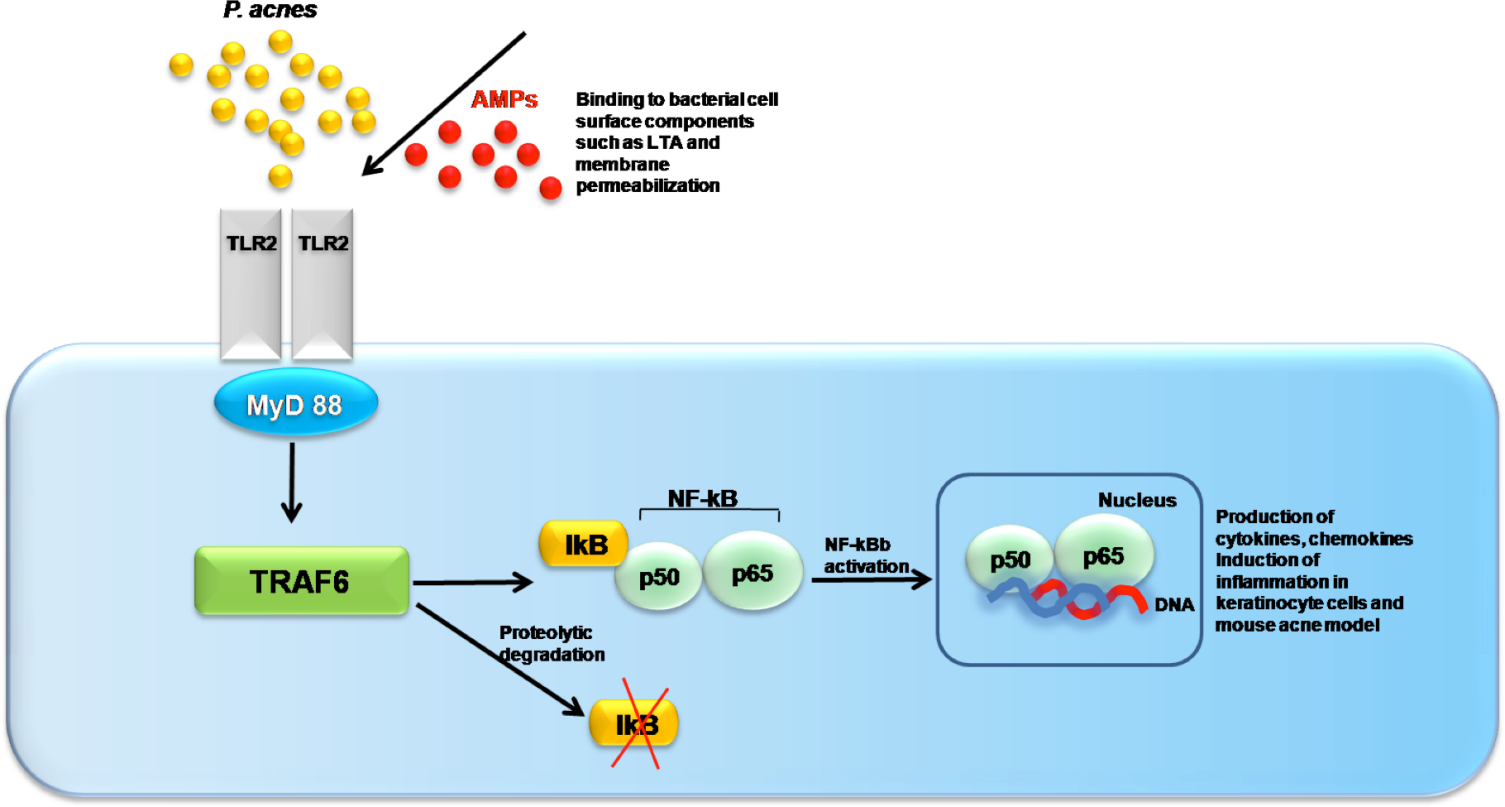
Schematic diagram of P5 based on the toll-like receptor-mediated immune response against *P*. *acnes*. Schematic diagram illustrating the proposed mechanism by which P5 suppresses *P*. *acnes*-induced inflammatory responses within acne lesions by disrupting TLR2-to-NF-κB signaling.

The major signaling pathway used by most TLRs involves the activation of the NF-κB, which in turn leads to the expression of various cytokines, chemokines, adhesion molecules and granulopoiesis factors, all of which would contribute to the cutaneous host defense against *P*. *acnes* [40]. Our results indicate that P5 effectively inhibits *P*. *acnes*-induced nuclear translocation of NF-κB in HKs (Fig 5). We therefore suggest that the interaction of P5 with both *P*. *acnes* cells and the TLR ligands they express prevents inflammation by inhibiting the TLR-to-NF-κB signaling cascade in keratinocytes (Fig 8).

Consistent with that idea, Guarna *et al*. recently suggested that cationic peptides suppress inflammatory actions in acne by binding to bacterial factors such as LTA and inhibiting host cell secretion of pro-inflammatory cytokines [41]. Although the mechanism by which cationic AMPs act to modulate host cell responses is not fully understood, it is thought that direct binding of the peptides to pathogen-associated molecular patterns (PAMPs) may play a role [42]. Such binding would reduce the number of PAMPs available to interact with their receptors, thereby preventing host cell activation. In addition, Scott *et al*. showed that a variety cationic AMPs block LPS binding to LPS-binding protein, and that this activity correlated with the ability to inhibit LPS-induced TNF-α production [29]. Based on these earlier studies, we analyzed CD spectra to determine whether P5 would bind purified LTA *in vitro*. When P5 was incubated at 50 μM in a 0.1% LTA suspension, the peptide adopted a folded conformation, which is indicative of its binding to LTA (S1 Fig). Thus P5’s anti-inflammatory activity in HKs appears to be associated, at least in part, with its binding to bacterial cell wall components, like LTA. These results also suggest that the anti-inflammatory activity of P5 may be attributable in part to its ability to neutralize pro-inflammatory bacterial factors through interaction with various PAMPs.

Ca^2+^ signaling appears to be involved at several stages of bacterial infection, among which the expression and secretion of pro-inflammatory mediators and cytoskeletal reorganization are of particular relevance during host cell infection by bacterial pathogens. By evoking Ca^2+^ signaling, bacterial toxins induce expression of various genes, including those encoding IL-6, IL-8 and mucin [43]. Recent studies also indicate TLR signaling can stimulate Ca^2+^ mobilization [25, 44] that plays a key role in pro-inflammatory cytokine production (TNF-α and IL-6) induced via the PGN/TLR2 and LPS/TLR4 pathways [45]. In addition, Katsuta *et al*. showed that free fatty acids are released from triglycerides in the sebum by *P*. *acnes* in the hair canal and on the skin surface, and that components of sebum alter intracellular free Ca^2+^ levels in epidermal keratinocytes, thereby inducing the abnormal keratinization involved in comedo formation in acne vulgaris [46]. Moreover, an influx of Ca^2+^ into epidermal keratinocytes reportedly delays recovery of skin barrier function, leading to epidermal hyperplasia [47]. Our results showed that *P*. *acnes* induces intracellular Ca^2+^ fluctuations in HKs *in vitro* and that P5 significantly inhibits that response (Fig 6). This suggests P5 may block the *P*. *acnes*-induced Ca^2+^ signaling that leads to the abnormal follicular keratinization and comedo formation characterizing acne vulgaris.

Within acne lesions, an increase of *P*. *acnes* numbers evokes an inflammatory response in the skin [48]. Similarly, injecting live *P*. *acnes* into the skin leads to the development of inflammatory skin disease in animal models [49]. *P*. *acnes* colonizes the skin surface and sebaceous follicles, and overgrowing *P*. *acnes* can rupture the follicle wall, allowing the bacteria to enter the dermis [50], where they come in contact with the host immune system, causing granulomatous inflammation [51]. To evaluate the effects of P5 *in vivo*, we used a well-known animal model of acne, the ICR mouse, in which the pattern of *P*. *acnes*-induced inflammation is similar to that in human acne lesions [52]. We found that intradermal injection of live *P*. *acnes* into the mouse ear caused a thickening of the ear and a granulomatous response. Notably, intradermal injection of P5 provided protection against *P*. *acnes*-induced ear inflammation (Fig 7). P5 efficiently suppressed this *P*. *acnes*-induced inflammatory response the resultant ear thickening (Fig 7B), and correspondingly reduced the bacterial load within the ear tissue (Fig 7C). Thus P5 appears to act effectively against *P*. *acnes in vivo* in the ICR mouse ear model.

Our findings indicate P5 is highly active against *P*. *acnes in vivo*. Up to now, translating AMP activity from *in vitro* experimentation to clinical application has been difficult due to their high systemic toxicity, instability, and rapid inactivation and degradation *in vivo*. In this study, however, we describe a newly designed, safe and effective AMP that appears to have high potential for use against bacterial skin infections.

## Conclusion

A key factor involved in the pathophysiology of acne vulgaris is the presence of *P*. *acnes*, which triggers inflammation in acne sufferers through activation of the innate immune receptor TLR2 on keratinocytes. One effective treatment strategy for acne is the reduction of *P*. *acnes* in the skin. Endogenous AMPs such as β-defensin are able to directly kill *P*. *acnes* and modulate interactions between the innate and adaptive immune systems. In many individuals, however, the effects of endogenous agents do not reduce *P*. *acnes* sufficiently to prevent the pathogenesis of acne vulgaris. In the present study we examined the actions of an exogenous synthetic peptide, P5, against *P*. *acnes* in the skin. P5 exerts direct antimicrobial effects against *P*. *acnes*, but has no cytotoxic effects on host skin cells. P5 also binds to LTA *in vitro*, significantly reducing expression of the inflammatory cytokines IL-8 and TNF-α through inhibition of TLR2-to-NF-κB signaling. And *in vivo*, P5 exerts both anti-inflammatory and antimicrobial effects in a mouse model of *P*. *acnes* skin infection. Taken together, these findings suggest that P5 could potentially serve as an effective therapeutic agent for the treatment of acne vulgaris.

## Supporting Information

S1 Fig CD spectroscopic analysis of P5 binding to LTA. CD spectra for 50 μM CA-MA (solid line) and P5 (dotted line) were measured in the presence of 0.1% LTA.
(TIF)

S1 Table HK viability measured using MTT assays in the presence of synthetic AMPs. (TIF)
