# Supplementary figures and images for: Suppression of Propionibacterium acnes Infection and the Associated Inflammatory Response by the Antimicrobial Peptide P5 in Mice
Source: PLoS One. 2015 Jul 21;10(7):e0132619. doi: 10.1371/journal.pone.0132619 (PMC4510379; doi:10.1371/journal.pone.0132619)

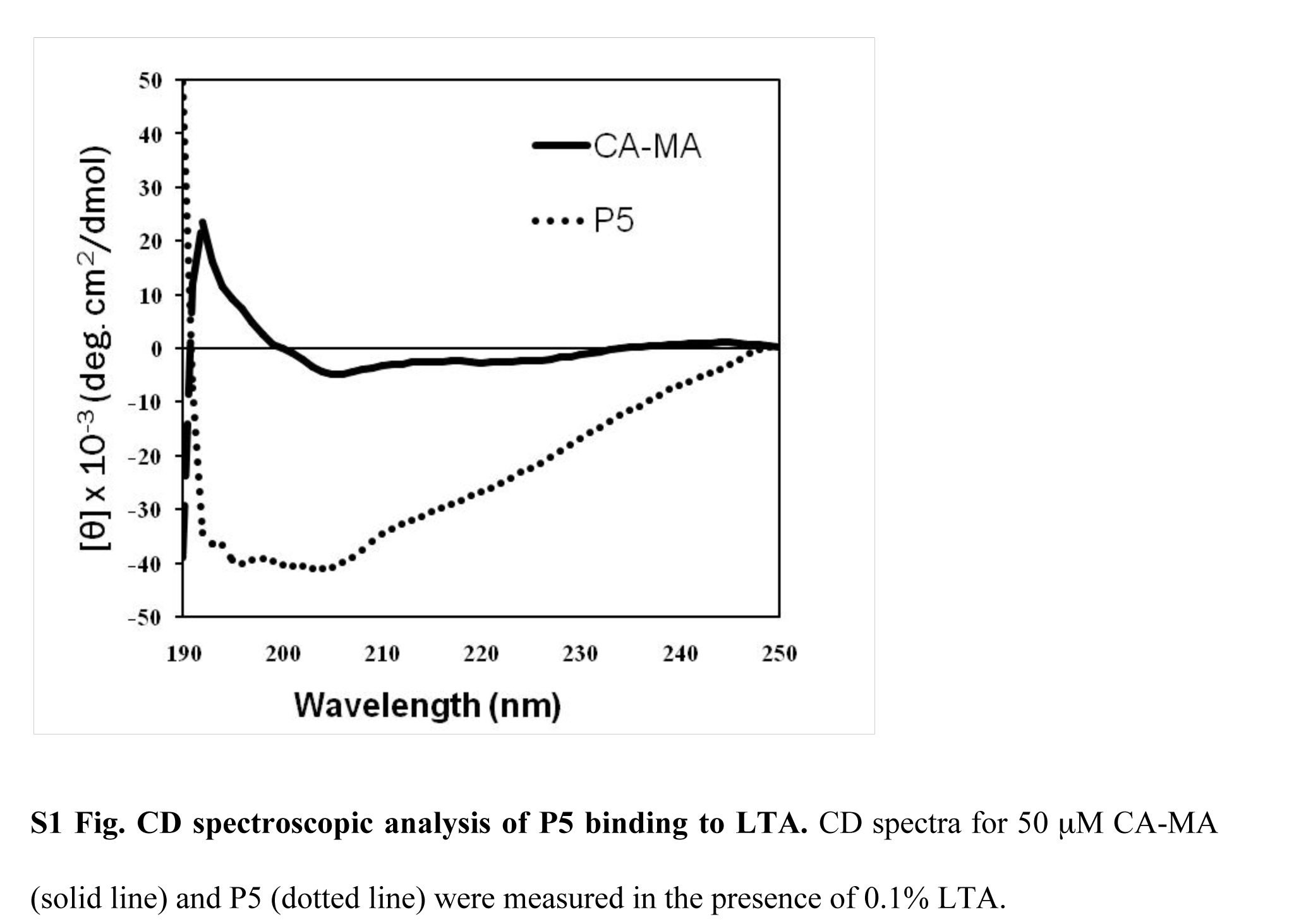

Supplement: S1 Fig — CD spectra for 50 μM CA-MA (solid line) and P5 (dotted line) were measured in the presence of 0.1% LTA. (TIF) [file pone.0132619.s001.tif]

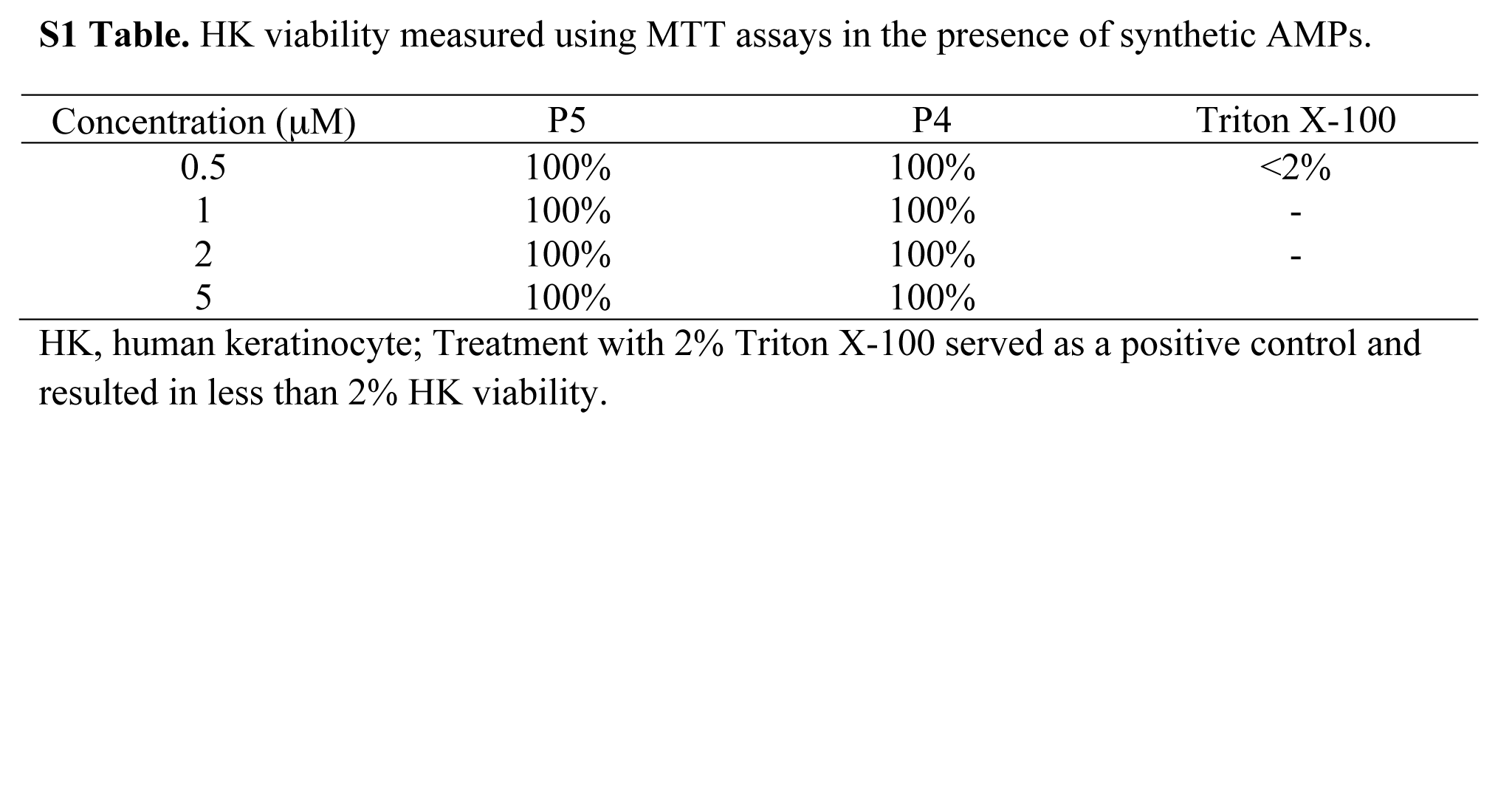

Supplement: S1 Table — (TIF) [file pone.0132619.s002.tif]
